# Medicaid CGM Barriers in Blind/Low Vision and Deaf/Hard of Hearing Populations

**DOI:** 10.1155/jdr/3621409

**Published:** 2026-05-08

**Authors:** Michelle L. Litchman, Andrew P. Bray, Jacee S. Moore, Chinue Wilford, Allyson S. Hughes

**Affiliations:** ^1^ College of Nursing, University of Utah, Salt Lake City, Utah, USA, utah.edu; ^2^ Ohio University Heritage College of Osteopathic Medicine, Athens, Ohio, USA, ohio.edu; ^3^ Department of Primary Care, Ohio University Heritage College of Osteopathic Medicine, Athens, Ohio, USA, ohio.edu

## Abstract

Continuous glucose monitors (CGMs) are essential tools for diabetes management; however, many state Medicaid programs do not have standardized, inclusive policies that ensure equitable access to CGMs for blind/low vision (BLV) and deaf/hard of hearing (DHH) populations, driving disparities across the United States and its territories. This policy analysis examines the barriers and facilitators to Medicaid CGM coverage for BLV and DHH populations, specifically in Medicaid and non‐Medicaid expansion states, and patient choice in CGM device selection. CGM barrier language existed in 9.6% of states and U.S. territories, preventing BLV and DHH populations from accessing CGM. Facilitator language was present in 5.8% of states and U.S. territories, advocating for equitable access to CGMs for BLV individuals but not DHH individuals. Given the limitations of CGM alarms, this paper also emphasizes the need for Medicaid to cover secondary devices that provide needed haptic, visual, and audible alarms as necessary accessibility tools rather than optional add‐ons. These devices are integral to ensuring a safe and independent CGM use for BLV and DHH individuals. Policy changes that prioritize health equity, framing CGM access as both a usability issue and a matter of eligibility for all Medicaid beneficiaries, is crucial.

## 1. Introduction

Continuous Glucose Monitoring (CGM) systems are integral in diabetes management, offering real‐time insights into glucose levels and trends to the 2.4 million CGM users in the United States.[1] Benefits include improved glycemic management by increasing time in range and decreasing A1C, [2] less hypoglycemic events, [3] higher quality of life, [4] and reduced risk of developing retinopathy. [5] However, those living with disability have barriers to CGM. In the United States, more than one in four people (28.7%) are disabled [6] and many rely on Medicaid for healthcare coverage [7]. Despite this need, people with disabilities in the United States face systemic barriers to accessing basic and specialized healthcare needs. [8] People with diabetes who are blind/low vision (BLV) or are deaf/hard of hearing (DHH) experience significant health disparities and barriers to diabetes technology uptake that impact their diabetes management. [9] Healthcare provider bias, which is common in BLV and DHH individuals, drives health disparities.[10] Diabetes is a federally protected condition under the Americans with Disabilities Act.[11]

The relationship between diabetes and vision loss is bidirectional such that people with diabetes are more likely to develop vision loss and people with vision loss are more likely to develop type 2 diabetes. [12] Diabetes is the leading cause of blindness, primarily due to complications of diabetic retinopathy and macular edema. [12] There also appears to be a bidirectional relationship between being DHH and diabetes. DHH populations report higher rates of diabetes than hearing counterparts, and those who have diabetes are more likely to report hearing loss. [13] Genetic mutations leading to Wolfram syndrome, maternally inherited diabetes and deafness, Rogers syndrome, and Hermann syndrome also factor into coexisting DHH and diabetes [14].

Sensory differences related to vision and hearing can make diabetes management significantly more difficult. For those with vision loss, tasks like monitoring blood glucose levels, administering insulin, preparing meals, and attending medical appointments all require adaptations. [9] For those with hearing loss, challenging tasks may include a lack of ASL interpreters at medical appointments and for those whose primary language is ASL, reading diabetes handouts in English. [15] Many BLV and DHH individuals with diabetes rely on specialized equipment, training, and support services to maintain their health and independence. [9] Significant health disparities exist for people with vision loss, such as lower quality of life, less access to healthcare, and higher rates of diabetes mortality. [16] Low health literacy is common in DHH individuals due to a scarcity of health information in ASL and due to a lack of ASL qualified interpreters. [17, 18] In addition, individuals who are BLV and DHH also experience systematic and structural bias in healthcare settings, further exacerbating health disparities.[19]

Medicaid is for people with disabilities and/or people who have low household income. [20] The Medicaid budget is primarily federal funding and supplemented by individual states. Medicaid funding is crucial for people with diabetes, though disparities exist nationwide. Medicaid diabetes policies vary from state to state, creating a patchwork system that leaves individuals in some regions without access to diabetes technology. Additionally, financial constraints within Medicaid programs often result in lower prioritization of newer technologies, like CGM, forcing individuals to rely on less effective treatment options. Further complicating the issue are FDA limitations, which can restrict the use of certain diabetes technologies based on specific medical conditions or stages of the disease, and Medicaid may adhere to these restrictions, further limiting access. These barriers contribute to significant health inequities, as low‐income individuals who rely on Medicaid often face the greatest challenges in managing their diabetes effectively. To ensure that all individuals, regardless of income or location, have equal access to the advancements in diabetes care, policy reforms are needed to expand Medicaid coverage and remove these obstacles. Medicaid expansion (a provision under the Affordable Care Act) allows states to extend Medicaid coverage to more low‐income individuals, specifically those with incomes up to 138% of the federal poverty level. [21] Although Medicaid expansion has significantly increased healthcare access in many states, not all states have chosen to expand the program.

The 2025 *American Diabetes Association Standards of Care* emphasizes the importance of diabetes technology in improving outcomes and diabetes management, “The choice of CGM device should be made based on the individual′s circumstances, preferences, and needs.” [22] However, despite these standards, existing health policies, including those shaped by FDA limitations, create significant barriers to equitable access. These regulatory restrictions often disproportionately affect underserved populations, including those who are BLV and DHH, hindering their ability to benefit from advances in diabetes care technology and exacerbating healthcare disparities.

The purpose of this policy analysis was to (1) examine barriers and facilitators of state Medicaid coverage of CGM for BLV and DHH populations, (2) assess CGM barriers differences for BLV and DHH populations in Medicaid and non‐Medicaid expansion states, and (3) assess how state CGM policy aligns with American Diabetes Association recommendations to offer patient choice in diabetes technology selection.

## 2. Materials and Methods

### 2.1. Design

A comprehensive examination of Medicaid coverage for CGM coverage was conducted in all 50 states, Washington DC, and Puerto Rico. Publicly available Medicaid policies were coded by all the authors in a spreadsheet. Coded documents included policies or formularies on the state‐based Medicaid website and/or prior authorization documents. Coding occurred between February 21, 2025, and April 1, 2025.

### 2.2. Measures

#### 2.2.1. Diabetes Type

For each state, we captured CGM coverage data specific to each type of diabetes. This was coded as type 1, type 2, and gestational diabetes/pregnancy in diabetes.

#### 2.2.2. Accessibility Barriers for DHH and BLV Populations

Accessibility barrier includes language that was prohibitive of a DHH/BLV individual being able to obtain CGM that was separate from the standard policy.

#### 2.2.3. Accessibility Facilitators for DHH and BLV Populations

Accessibility factor includes language that was protective of a DHH/BLV individual being able to obtain CGM that was separate from the standard policy.

#### 2.2.4. CGM Device Coverage

Each state was coded based on coverage for the FDA‐approved CGM devices.

#### 2.2.5. Patient Choice for CGM Usage

Patient choice for CGM usage was assessed by coding for the number of devices offered amongst FDA‐approved CGM devices in the United States. A continuous number, based on how many devices each state provided coverage for, was used.

#### 2.2.6. Medicaid Expansion State

Each state was coded based on its Medicaid expansion status. At the time of coding, non‐Medicaid expansion states were Alabama, Florida, Georgia, Kansas, Mississippi, South Carolina, Tennessee, Texas, Wisconsin, and Wyoming.

#### 2.2.7. Analysis

Descriptive statistics are presented. Descriptive statistics were conducted using SPSS Version 28.

## 3. Results

### 3.1. Policy Barriers and Facilitators for CGM Use in DHH/BLV Populations

**Figure 1:**
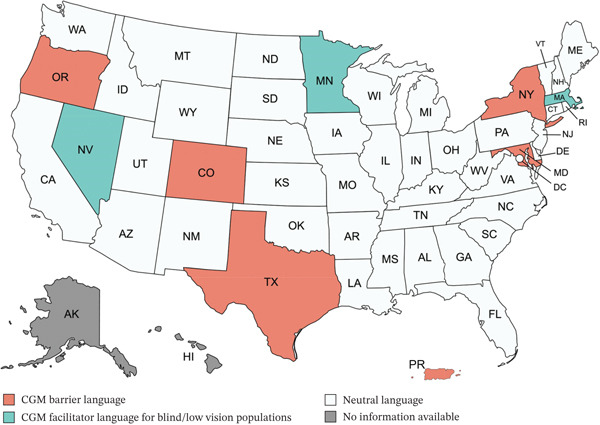
Barriers and facilitators to CGM use in blind/low vision and deaf/hard of hearing populations.

The majority of states did not note any barrier or facilitator language. Using neutral language, vision, or hearing was not addressed at all. Example neutral language included: “Can recognize and respond to the messages, alarms, and alerts of the device.”[23] Figure 1 graphically depicts barriers and facilitators across the states and U.S. territories.

There were 9.6% of states and U.S. territories coded as using policies that are barriers for diabetes technology for BLV and DHH populations. Language specifically noted a requirement of the person with diabetes and/or their caregiver be able to see or hear. Example barriers language included: “Be able to hear and view the CGM alerts and respond accordingly or have a caregiver who is able to do so.”[24] Table 1 shows the specific barrier and facilitator language to CGM use in DHH and BLV populations used by specific states.

**Table 1:** CGM barriers and facilitators language by state.

State	Deaf/hard of hearing populations	Blind/low vision populations
*Barriers to CGM*		
Colorado[25]	Be able to hear and view the CGM alerts and respond accordingly, or have a caregiver who is able to do so.	Be able to hear and view the CGM alerts and respond accordingly, or have a caregiver who is able to do so.
Maryland[26]	If a member chooses to purchase a CGM with additional or special features (e.g., talking device), the member will be responsible for the cost of the additional/special features in full. [client] will authorize coverage of these additional/special features only if they are listed in the prior authorization request and [client] determines that they are reasonable and medically necessary due to a member′s particular comorbidity (e.g., vision impairment). Specifically, there must be clear and compelling evidence that the added features are expected to directly contribute towards improvement in the member′s glycemic control or reduction in the incidence of hypoglycemia.	If a member chooses to purchase a CGM with additional or special features (e.g., talking device), the member will be responsible for the cost of the additional/special features in full. [client] will authorize coverage of these additional/special features only if they are listed in the prior authorization request and [client] determines that they are reasonable and medically necessary due to a member′s particular comorbidity (e.g., vision impairment). Specifically, there must be clear and compelling evidence that the added features are expected to directly contribute towards improvement in the member′s glycemic control or reduction in the incidence of hypoglycemia.
Mississippi[27]	Be able, or have a caregiver who is able, to hear and view CGM alerts and respond appropriately.	Be able, or have a caregiver who is able, to hear and view CGM alerts and respond appropriately.
New York[28]	Are able, or have a caregiver who is able, to hear and view CGM alerts and respond appropriately	Are able, or have a caregiver who is able, to hear and view CGM alerts and respond appropriately
Oregon[24]	Member or caregiver can hear and view CGM alerts and respond appropriately.	Member or caregiver can hear and view CGM alerts and respond appropriately.
*Facilitators to CGM*		
Massachusetts[29]	—	Providers may request an exception from the insulin use requirement for members not receiving insulin due to physical disability, visual impairment, documented needle phobia, cognitive impairment, or age.
Minnesota[30]	—	Approval criteria for nonpreferred products—Patient is legally blind or has reduced visual acuity so that they are unable to see the numbers on ALL of the preferred products and the requested product has a feature that enables the patient to use the product that is not available on any of the preferred products. The patient (not a caregiver) must be the one using the products.
Nevada	—	Recipient has a visual impairment that requires the use of a nonpreferred product.

Conversely, only 5.8% of states and U.S. territories were coded as using policies that facilitate diabetes technology uptake for the BLV and DHH populations. Massachusetts language focused on how providers could request an exemption for the insulin requirement in BLV individuals, whereas Minnesota and Nevada focused on language that allowed for BLV individuals to access a nonpreferred CGM device if it met their visual needs. See specific language in Table 1. There were no states that included facilitator language specific to DHH populations.

### 3.2. CGM Coverage in Medicaid Expansion States

There are 82.3% (*n* = 40) states and U.S. territories that have Medicaid expansion. Of these, five (12.5%) have barrier language, preventing CGM coverage to BLV and DHH individuals. All non‐Medicaid expansion states have language that allows for CGM coverage to BLV and DHH individuals.

### 3.3. Coverage of CGM Type by State and U.S. Territory

Patient choice for CGM coverage was 2.5 devices (SD 2.4, Range 0–7). The frequency in which states and U.S. territories offered CGM choices were as follows: 1.9% (*n* = 1) offered one choice, 7.7% (*n* = 4) offered two choices, 9.6% (*n* = 5) offered three choices, 13.5% (*n* = 7) offered four choices, 15.4% (*n* = 8) offered five choices, 9.6% (*n* = 5) offered six choices, and 1.9% (*n* = 1) offered seven choices. The CGM brands with the highest coverage were Dexcom G6 (56%) and Freestyle Libre 2 (48%). See Table 2 for additional coverage details.

**Table 2:** CGM coverage.

CGM type	Percentage of coverage
Dexcom G6	56%
Dexcom G7	38%
Eversense	0%
Freestyle Libre 14	27%
Freestyle Libre 2	48%
Freestyle Libre 3	46%
Freestyle Libre 3 PLUS	10%
Freestyle Libre PRO	2%
Guardian Connect	8%

## 4. Discussion

This policy analysis is the first to examine CGM coverage for BLV and DHH populations. Findings underscore significant disparities in access to CGM. These regional gaps are exacerbated by broader systemic inequities that disproportionately impact historically marginalized populations, including individuals who are BLV and DHH.

CGM coverage language in individual state policies vary. We found that 9.6% of states and U.S. territories create additional barriers to obtaining CGM in BLV and DHH populations. Whereas others have identified that bias does exist in CGM prescribing in certain populations due to systematic barriers [31, 32], this is the first paper to highlight how policy language has created exponential structural barriers to CGM use in BLV and DHH populations even if a prescription is present. Massachusetts, Nevada, and Minnesota provide facilitator language that specifically promotes CGM use in BLV populations, and their policies should be looked to as exemplars. Notable, however, facilitator language was lacking for DHH populations in all states. In the future, states should use more neutral language that focuses on whether the person with diabetes or their caregiver can recognize and respond to CGM messages, alarms, and alerts, rather than focusing on ability to see or hear.

Although this study focused on access to CGM via Medicaid, it is important to note other barriers that BLV and DHH face when using CGM that require resolution. Our prior work has shown that not all CGM alarms accommodate varied sensory needs.[9] For example, while CGM instructions are now available in several written languages, instructions remain unavailable in Braille and ASL. Additionally, audible alerts do not accommodate the needs of many DHH individuals, visual and haptic (vibration) alarms are required. However, many CGM devices do not provide visual or haptic alarms without secondary devices (e.g., smart phone and smart watch) that is not covered by Medicaid. As such, CGM devices are not “ready out of the box” for BLV and DHH individuals furthering the potential for disparities. Medicaid should provide coverage for these necessary secondary devices that allow CGM to be more accessible.

Given the spectrum of vision in someone who is BLV or hearing in someone who is DHH, individual circumstances, preferences, and needs—patient choice—should be addressed in CGM selection. Patient choice, endorsed by the American Diabetes Association′s 2025 Standards of Care[22] increases the likelihood of CGM usage and functionality. We found that states offered at least two CGM options 98.1% of the time. Although this is promising, it is important to note that each CGM device offers features that will benefit individuals differently, based on their accessibility needs, and the preferred choices may not necessarily match the BLV or DHH individual needs. Endocrinologists surveyed about their BLV patients reported that CGM with voice activation did reduce A1C and frequency of hypoglycemia.[33] A CGM device with voice activation is likely to yield the most glycemic improvement; however, not all CGM devices provide this feature. Nonpreferred devices were offered in only two states for those with BLV. Unfortunately, DHH populations have been completely left out of any CGM facilitator language, and therefore, patient choice, despite their individual circumstances, does not address preferences and needs.

The goals of this paper were to conduct a policy analysis of current Medicaid policy as it relates to diabetes management for BLV and DHH people. Though not examined in this paper, it is important to note that other types of disabilities (cognitive, mobility, etc.) should be included in state Medicaid diabetes coverage policies. Current eligibility criteria for CGM often fail to account for accessibility needs, and in some cases, explicitly or implicitly exclude BLV and DHH individuals. The exclusion of people from CGM access on the basis of visual or hearing status is structural ableism—discrimination rooted in the assumption that only certain bodies or abilities are “normal” or acceptable for diabetes technology use. Although typically Medicaid expansion promotes health equity, we found barriers in some Medicaid expansion states and none in nonexpansion states. This needs to be immediately examined and addressed so all people who need CGM have access to this technology.

As federal and state administrations consider slashing Medicaid budgets for people with existing health conditions, including people with diabetes who are BLV and DHH, CGM access may be challenged. We urge policymakers, pharmaceutical and device manufacturers, insurers, and healthcare providers to take concrete steps toward ensuring equitable access to diabetes technology to prevent untoward harm. Steps include designing and distributing CGMs that are accessible for people with disabilities, supporting CGM instructions in ASL and Braille, and advocating for coverage reforms that prioritize inclusion and accessibility over cost or perceived utility. Addressing ableism in diabetes care is not only a legal and ethical imperative—it is essential for advancing health equity.

## 5. Conclusion

Medicaid coverage for advanced technologies, such as CGM, is often limited or inconsistent, with many states not fully covering these devices or requiring strict documentation and prior authorization. This disparity is exacerbated by the fact that Medicaid policies vary from state to state, creating a patchwork system that leaves individuals in some regions without access to critical tools for managing their diabetes. Regardless of disability status, CGM should be accessible to all.

## Funding

No funding was received for this manuscript.

## Conflicts of Interest

The authors declare no conflicts of interest.

## Data Availability

The data that support the findings of this study are available on request from the corresponding author. The data are not publicly available due to privacy or ethical restrictions.
